# Energy Transfer between Conjugated Colloidal Ga_2_O_3_ and CdSe/CdS Core/Shell Nanocrystals for White Light Emitting Applications

**DOI:** 10.3390/nano6020032

**Published:** 2016-02-15

**Authors:** Paul C. Stanish, Pavle V. Radovanovic

**Affiliations:** Department of Chemistry, University of Waterloo, 200 University Avenue West, Waterloo, ON N2L 3G1, Canada; pstanish@uwaterloo.ca

**Keywords:** Förster resonance energy transfer, colloidal nanocrystals, white light, photoluminescence spectroscopy, donor, acceptor, gallium oxide, II–VI semiconductor, time-resolved photoluminescence spectroscopy

## Abstract

Developing solid state materials capable of generating homogeneous white light in an energy efficient and resource-sustainable way is central to the design of new and improved devices for various lighting applications. Most currently-used phosphors depend on strategically important rare earth elements, and rely on a multicomponent approach, which produces sub-optimal quality white light. Here, we report the design and preparation of a colloidal white-light emitting nanocrystal conjugate. This conjugate is obtained by linking colloidal Ga_2_O_3_ and II–VI nanocrystals in the solution phase with a short bifunctional organic molecule (thioglycolic acid). The two types of nanocrystals are electronically coupled by Förster resonance energy transfer owing to the short separation between Ga_2_O_3_ (energy donor) and core/shell CdSe/CdS (energy acceptor) nanocrystals, and the spectral overlap between the photoluminescence of the donor and the absorption of the acceptor. Using steady state and time-resolved photoluminescence spectroscopies, we quantified the contribution of the energy transfer to the photoluminescence spectral power distribution and the corresponding chromaticity of this nanocrystal conjugate. Quantitative understanding of this new system allows for tuning of the emission color and the design of quasi-single white light emitting inorganic phosphors without the use of rare-earth elements.

## 1. Introduction

Colloidal nanocrystals (NCs) offer a substantial promise for the design and fabrication of solid state structures and devices. Size-tunable electronic structure and optical properties render this class of materials attractive for various applications in photonics, optoelectronics, and sensors [1]. The size and shape of colloidal NCs can be controlled *in situ* by simply adjusting the synthesis conditions [2], allowing for the exploitation of quantum confinement to manipulate their optical properties. Furthermore, colloidal NCs can be modified post-synthetically by functionalization and conjugation with organic or biomolecules [3], or by forming a composite with polymers or other nanostructures [4,5]. This ability to combine solid state-like optical and mechanical properties of NCs with the opportunities for their chemical modifications in solution opens a number of possibilities to use them as building blocks in optical and photonic devices.

With increasing concerns about the global energy supply and sustainable use of natural resources, the search for new low-cost materials for energy efficient lighting has intensified in recent years [6]. The total amount of electrical energy used for lighting exceeds 20% at the global level, with residential and commercial sectors being responsible for the majority of lighting electricity consumption [7]. Light emitting diodes (LEDs) have emerged as a long-term alternative to traditional incandescent light bulbs, owing to their efficiency, durability, and reliability [8]. The most promising approach to white LEDs has been to combine GaN blue LED with remote phosphors, most notably yellow-emitting yttrium aluminum garnet doped with cerium (Ce:YAG) [9]. However, the adoption of such white LEDs for general lighting applications has been rather slow because of the high manufacturing cost, and non-optimal characteristics of light (low color rendering index and high correlated color temperature) [10]. The deficiencies of the spectral properties have been addressed by the addition of other rare earth element-based phosphors to augment the spectral density in the red part of the spectrum [11,12], further increasing the complexity and cost of the final devices and making it difficult to obtain homogeneous white light in a reproducible manner. Colloidal II–VI NCs with selected average sizes have also been used in combination with remote phosphors to generate “warm” white light [13].

We have recently demonstrated the ability to produce white light and tune its chromaticity by conjugating transparent metal oxide NCs with organic fluorophores (dyes) emitting in the complementary spectral range [14,15]. The core of this phenomenon is a defect-based photoluminescence of these metal oxide NCs. In the case of colloidal γ-Ga_2_O_3_ NCs the emission is based on electron donor-acceptor pair (DAP) recombination [16]. It arises from the recombination of an electron trapped on an oxygen vacancy forming localized electron donor states with a hole trapped on a gallium–oxygen vacancy pair acting as an electron acceptor [17,18]. A range of distances between electron donor and acceptor sites, as well as coupling of the electronic transition with lattice phonons are likely reasons for significant broadening of this emission band [17,19]. Owing to the proximity of the NC surface-bound organic fluorophore to the DAP recombination sites, and the overlap of its absorption spectrum with the DAP photoluminescence (PL) band, the photoexcited Ga_2_O_3_ NCs can transfer the excitation energy to the conjugated dye molecules via the Förster resonance energy transfer (FRET) mechanism [20]. This approach has allowed us to obtain a hybrid nanoconjugate acting as a single white light-emitting fluorophore [11,12].

In this work, we demonstrate the design and preparation of new colloidal white light-emitting nanocomposite obtained by controlled conjugation of II–VI and γ-Ga_2_O_3_ NCs using a simple molecular linker. The ability to manipulate the PL spectra of CdSe/CdS (core/shell) NCs by changing the core size and/or shell thickness allows for fine tuning of the red side of the nanocomposite emission to generate white light with the desired chromaticity. The spectral overlap between the PL of the energy donor (Ga_2_O_3_ NCs) and the absorption of the energy acceptor (core/shell CdSe/CdS NCs) enables their electronic coupling by FRET. Using the PL quenching of the energy donor determined from the steady-state and time-resolved PL measurements, we quantified the contributions of the direct excitation and energy transfer to the emission of CdSe/CdS NCs. The nanocomposite demonstrated in this work represents an important step toward single-phased all-inorganic rare earth element-free white light-emitting phosphor.

## 2. Results and Discussion

**Figure 1:**
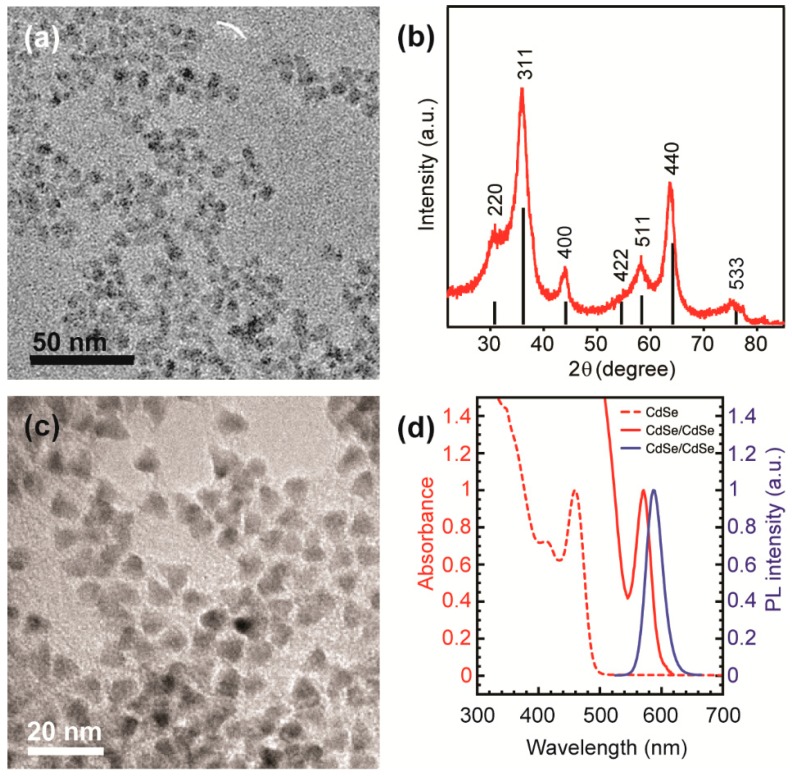
(**a**) Overview transmission electron microscopy (TEM) image of colloidal γ-Ga_2_O_3_ nanocrystals (NCs) having an average dimeter of *ca*. 5.5 nm; (**b**) X-ray diffraction (XRD) pattern of the same NCs; vertical black lines represent a reference XRD pattern of bulk γ-phase Ga_2_O_3_; (**c**) Overview TEM image of *ca*. 6.8 nm CdSe/CdS core/shell NCs; (**d**) Absorption (red) and photoluminescence (PL) (blue) spectra of CdSe/CdS NCs (solid lines), and the absorption spectrum of the corresponding CdSe NC cores (red dashed line).

The average size and morphology of colloidal Ga_2_O_3_ NCs used in this study were determined by transmission electron microscopy (TEM). The NCs are quasi-spherical and have an average diameter of *ca.* 5.5 nm (Figure 1a). The X-ray diffraction (XRD) pattern confirms that these NCs exhibit cubic crystal structure characteristic for the γ-Ga_2_O_3_ (Figure 1b). We chose CdSe/CdS core/shell NCs as a complementary solid-state emitter because they possess a strong size-tunable red emission, which could allow for the generation of white light when coupled with the Ga_2_O_3_ DAP PL. A typical TEM image of CdSe/CdS NCs is shown in Figure 1c. As-synthesized CdSe NC cores have an average size of *ca*. 2.2 nm, as determined from the band gap absorption energy [21]. From the comparison of the average NC sizes before and after the shell growth, the average thickness of the shell was estimated to be *ca.* 2.3 nm, consistent with the core/shell NCs prepared under similar conditions [22]. These estimates were confirmed using the corresponding band edge absorption energy (Figure 1d).

**Figure 2:**
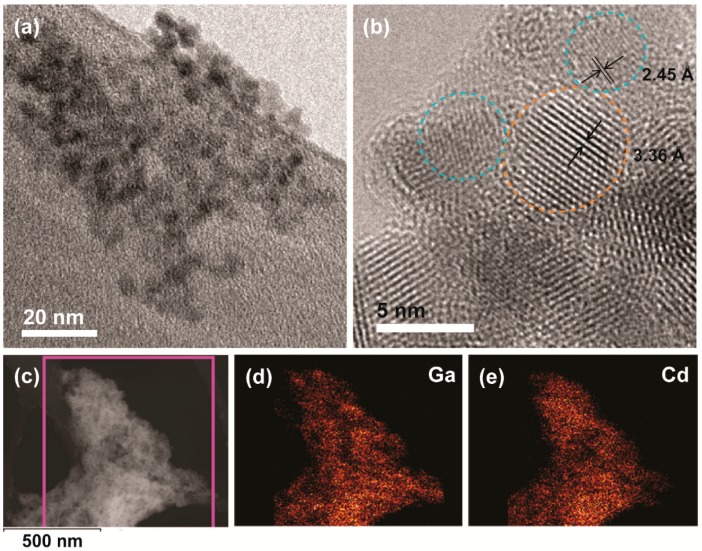
(**a**) Overview TEM image of colloidal Ga_2_O_3_-CdSe/CdS NC conjugate obtained using NCs shown in Figure 1; (**b**) High resolution TEM image of the same sample. Orange and blue circles indicate CdSe/CdS and Ga_2_O_3_ NCs, respectively; the lattice spacings shown correspond to {002} and {311} planes of CdS and Ga_2_O_3_, respectively; (**c**–**e**) Scanning transmission electron microscopy (STEM) image of the nanocrystal conjugate (**c**); and the corresponding Ga (**d**) and Cd (**e**) energy-dispersive X-ray spectroscopy (EDX) elemental maps.

To facilitate the conjugation of Ga_2_O_3_ and CdSe/CdS NCs we used thioglycolic acid (TGA), which can bind to NC surfaces through both thiol and carboxylic acid functional groups [23]. Thiols are known to bind strongly to the CdSe or CdS quantum dot surfaces [24,25], and in our previous studies we showed that carboxylic acid group reacts with the surface of Ga_2_O_3_ NCs [15,20]. An overview TEM image of the CdSe/CdS-conjugated γ-Ga_2_O_3_ NCs is shown in Figure 2a. The NCs form extended networks owing to the strong binding of the linker to the NC surfaces. Figure 2b shows a high resolution TEM image of conjugated NCs. The nanocrystals within a formed aggregate are identified by measuring their lattice spacing. The close proximity of Ga_2_O_3_ and CdSe/CdS NCs shown in Figure 2b confirm their conjugation. To determine the overall homogeneity of the colloidal NC conjugate we performed energy-dispersive X-ray spectroscopy (EDX) elemental mapping in the scanning transmission electron microscopy (STEM) mode. A STEM image and the corresponding maps of Ga and Cd are shown in Figure 2c–e, respectively. The Ga and Cd maps are well correlated, indicating homogeneous distribution of both types of NCs within the aggregate.

**Figure 3:**
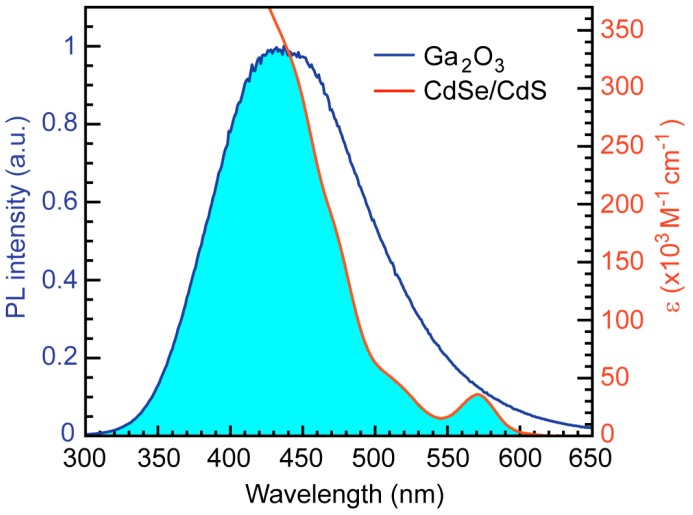
Absorption spectrum of CdSe/CdS NCs (orange line) and PL spectrum of Ga_2_O_3_ NCs (blue line). Excitation wavelength for Ga_2_O_3_ NCs is 250 nm. Shaded area indicates a spectral overlap, as an essential requirement for Förster resonance energy transfer (FRET).

CdSe/CdS NCs are excellent FRET acceptors because their absorption spectrum strongly overlaps with the emission spectrum of Ga_2_O_3_ NCs (Figure 3), providing a basis for the FRET mechanism. Furthermore, the quantum yield of these NCs is very high, in some cases reaching over 90% [26]. The spectral overlap is calculated as:

(1) $$J\left(\lambda \right)=\int_{0}^{\infty }F_{D}\left(\lambda \right)\varepsilon_{A}\left(\lambda \right)\lambda^{4}d\lambda$$

where *F*_D_(λ) is the emission of the FRET donor with an integrated intensity normalized to unity, and ε_A_(λ) is the molar extinction coefficient of the acceptor at wavelength λ. Spectral overlap is the key parameter determining the critical radius (*R*_0_), also known as the Förster radius, which is defined as the separation at which the energy transfer efficiency is 50%.

(2) $$R_{0}={\left(\frac{9000\left(\ln10\right)\kappa^{2}Q_{0}}{128\pi^{5}N_{A}n_{r}^{4}}J\left(\lambda \right)\right)}^{\frac{1}{6}}$$

In this expression *Q*_0_ is the donor quantum yield in absence of the acceptor, *n*_r_ is the index of refraction of the solvent, *N*_A_ is the Avogadro's number and κ^2^ is the dipole alignment factor which we define as 2/3. This value is appropriate when donor and acceptor dipole orientations are random and experience no ordering [20,27,28]. In the case of Ga_2_O_3_ and CdSe/CdS NCs, the Förster radius was calculated to be 7.13 nm. In order to observe FRET in colloidal suspension, Ga_2_O_3_ and CdSe/CdS NCs must be within 21.4 nm (3*R*_0_). This distance is much smaller than the average separation of free standing NCs in solution phase, which was estimated to be over 250 nm even for the highest NC concentrations used. To induce FRET in solution, the NCs were conjugated with a bifunctional TGA linker, as described in the Experimental Section. Based on the TEM images, the average sizes of Ga_2_O_3_ and CdSe/CdS NCs are *ca.* 5.5 and 6.8 nm, respectively. Assuming the length of TGA of 0.5 nm, the center-to-center separation between bound NCs should be *ca*. 6.7 nm, well within the limit of 21.4 nm.

**Figure 4:**
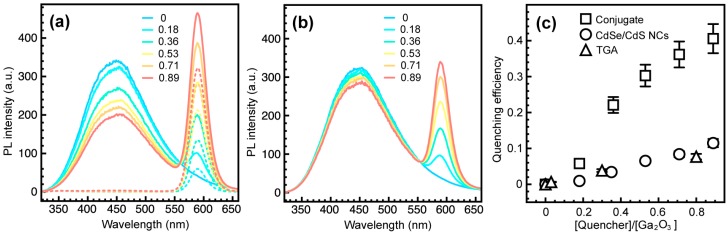
(**a**) PL spectra of colloidal Ga_2_O_3_-CdSe/CdS NC conjugates (solid lines) having different CdSe/CdS to Ga_2_O_3_ NC concentration ratio, as indicated in the graph. PL spectra of thioglycolic acid (TGA)-bound CdSe/CdS NC suspensions having the same concentration as in the NC conjugate are shown with dashed lines (λ_exc_ = 250 nm); (**b**) PL spectra of the mixtures of Ga_2_O_3_ and CdSe/CdS NCs prepared in the same way as the NC conjugate but without the TGA linker; (**c**) Quenching efficiency of the donor-acceptor pair (DAP) emission of Ga_2_O_3_ NCs in the conjugate (squares), mixed with CdSe/CdS NCs (circles), and mixed with TGA but without CdSe/CdS NCs (triangles).

Figure 4a shows PL spectra of Ga_2_O_3_-CdSe/CdS NC conjugates having different CdSe/CdS to Ga_2_O_3_ NC concentration ratio, upon excitation at 250 nm (corresponding to the Ga_2_O_3_ band edge). The concentration of CdSe NC cores was determined from the absorption spectrum using the extinction coefficient (ε) value reported for the samples prepared under similar conditions [21], which allowed us to estimate the concentration of CdSe/CdS NCs as FRET acceptors by assuming quantitative extraction and uniform coating of the cores with CdS shell. The relative concentrations of Ga_2_O_3_ NCs in the conjugate samples were inferred from EDX elemental analysis, accounting for an average NC volume. The emission intensity of Ga_2_O_3_ NCs is quenched up to *ca*. 40% concurrently with an increase in CdSe/CdS NC emission. Deconvoluted spectra of CdSe/CdS NCs in the nanocrystal conjugates are shown in Figure S1 (Supplementary Materials). This observation is consistent with the FRET coupling of the two components. However, CdSe/CdS NCs also emit light upon excitation at 250 nm. The PL spectra of CdSe/CdS NCs, treated exactly as in the preparation of NC conjugates but without Ga_2_O_3_ NCs, are shown with dashed lines in Figure 4a. Higher PL intensity of CdSe/CdS NCs in the conjugated form attests to FRET between Ga_2_O_3_ and CdSe/CdS NCs (Figure S1, Supplementary Materials). In contrast to the NC conjugate, a suspension containing the same concentrations of Ga_2_O_3_ and CdSe/CdS NCs without the linker exhibits only about a 10% decrease in Ga_2_O_3_ DAP emission intensity (Figure 4b). This reduction in the DAP emission occurs because of the direct excitation of CdSe/CdS NCs by a fraction of the excitation source, and is an unavoidable phenomenon when studying FRET between nanoparticles. Similarly, a solution containing Ga_2_O_3_ NCs and the relevant amount of TGA linker (*vide infra*) leads to only a 7.5% reduction in the DAP emission. To determine the concentration of TGA bound to the surface of CdSe/CdS NCs, the absorbance of TGA in the supernatant obtained upon precipitation of TGA-bound NCs was measured. After the initial wash *ca.* 94.5% of TGA remained in the supernatant. Additional washing resulted in further removal of the TGA loosely adsorbed on the NC surfaces, suggesting that only *ca.* 0.3% of the original amount of TGA is actually bound to CdSe/CdS NCs. Hereafter, the concentration of TGA is given in terms of the equivalents of CdSe/CdS NCs added to Ga_2_O_3_ NCs (*i.e.*, the amount of TGA corresponding to certain acceptor to donor concentration ratio). It is evident that some of the reduction in Ga_2_O_3_ PL intensity also comes from competitive light absorption and/or quenching by the TGA linker (Figure S2, Supplementary Materials), but the extent to which Ga_2_O_3_ NC emission is quenched by energy transfer is far greater (Figure 4c). The existence of FRET is also evident from the excitation spectrum of CdSe/CdS acceptor in the NC conjugate (Figure S3, Supplementary Materials).

**Figure 5:**
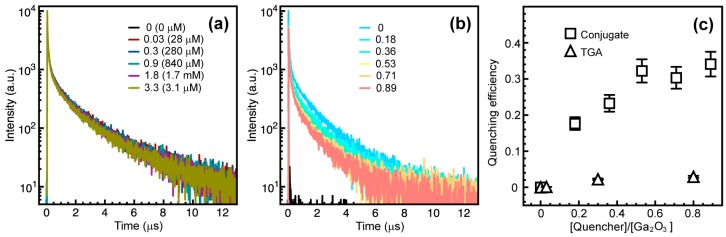
(**a**) Time-resolved DAP PL decay of Ga_2_O_3_ NCs containing different amounts of surface-bound TGA without CdSe/CdS NCs. TGA equivalents and absolute concentrations (in parentheses) in Ga_2_O_3_ NC suspensions are indicated in the graph (aqua blue trace (0.84 mM) represents TGA concentration for the highest acceptor to donor ratio); (**b**) Time-resolved DAP PL decay of Ga_2_O_3_ NCs in the NC conjugates having different CdSe/CdS to Ga_2_O_3_ NC concentration ratio, as indicated in the graph; (**c**) Quenching efficiency of the DAP PL of Ga_2_O_3_ NCs in the NC conjugate (squares) and with bound TGA (triangles).

The energy transfer efficiency is defined by the Förster theory as:

(3) $$\eta =\frac{nR_{0}^{6}}{nR_{0}^{6}+R_{\text{DA}}^{6}}$$

where *R*_DA_ is the average separation between donors and acceptors, and *n* is the acceptor to donor ratio. FRET efficiency (η) can also be determined experimentally by measuring the donor lifetime shortening in the presence of the acceptors:

(4) $$\eta =1-\frac{\tau_{\text{DA}}}{\tau_{D}}$$

where τ_D_ and τ_DA_ are the lifetimes of the donor alone and in the presence of the acceptors, respectively. Since DAP emission does not follow a simple exponential time decay [29], the integrated lifetimes were used to calculate η, instead of the time components obtained as the fitting parameters. The ligands bound to the Ga_2_O_3_ NC surface can compete with native defects for trapping of the photogenerated free carriers [14]. Therefore, the DAP emission can also be affected by the molecules bound to the surface of the NCs. When TGA replaces TOPO on Ga_2_O_3_ NCs in the amount equivalent to that used to conjugate CdSe/CdS NCs, the DAP emission experiences a negligible reduction in lifetime (Figure 5a). The original DAP PL lifetime is decreased by maximum of *ca*. 3% for the amount of TGA corresponding to the highest CdSe/CdS to Ga_2_O_3_ NC ratio explored in this study (aqua blue trace). On the other hand, Figure 4b shows progressive shortening of the Ga_2_O_3_ NC PL lifetime with increasing concentration of TGA-bound CdSe/CdS NCs, confirming that FRET is the dominant quenching mechanism. Figure 5c compares the quenching efficiency (η) of Ga_2_O_3_ NCs conjugated with CdSe/CdS NCs via TGA with that of Ga_2_O_3_ NCs capped with TGA, for different concentrations of the corresponding quencher, calculated using Equation (4). CdSe/CdS-conjugated Ga_2_O_3_ NCs show significantly higher efficiency for all quencher concentrations confirming the FRET from Ga_2_O_3_ donor to CdSe/CdS acceptor NCs.

**Figure 6:**
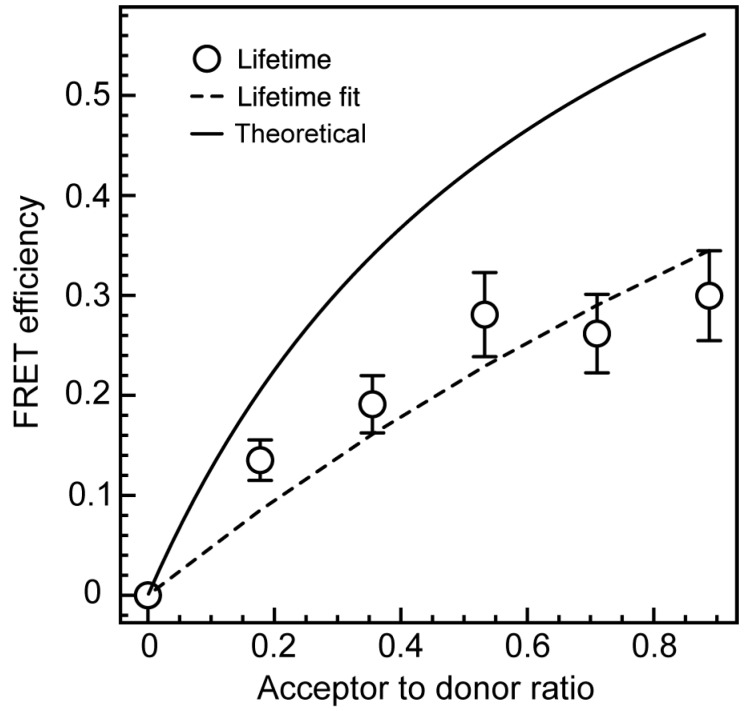
FRET efficiency in Ga_2_O_3_-CdSe/CdS NC conjugate (circles) determined from the DAP PL lifetime data corrected for the lifetime shortenening due to TGA binding. The experimental data were fit using Equation (5) (dashed line). Solid line is the FRET efficiency predicted by the Förster theory (Equation (3)).

Förster resonance energy transfer efficiency between Ga_2_O_3_ and CdSe/CdS NCs, calculated using Equation (4), was corrected for possible quenching of the donor emission by TGA (triangle symbols in Figure 5c), and displayed in Figure 6 as a function of the acceptor to donor ratio (*n*). The acceptor to donor ratio was determined to be 0.9 when 1 mL of CdSe/CdS NC suspension (the largest amount) was added to 1 mL of Ga_2_O_3_ NC stock suspension. The expression for FRET efficiency (Equation (3)) is an average approximation when the majority of binding sites are occupied or the stoichiometric ratio of the donor and acceptor can be controlled [20,28,30]. The colloidal NCs generally have a large surface area, leading to a large number of possible binding sites, some of which are unoccupied, invalidating this basic model. Instead, the distribution of the acceptors per donor will obey Poissonian statistical model when most binding sites are unoccupied [31,32]:

(5) $$\eta (\xi ,R)=\sum_{n=0}^{\infty }\frac{\xi^{n}e^{-\xi }}{n!}\left[\frac{nR_{0}^{6}}{R_{\text{DA}}^{6}+nR_{0}^{6}}\right]$$

where ξ is an average number of acceptors per donor, while *n* is a whole-number acceptor to donor ratio. The FRET efficiency data in Figure 6 were fit using Equation (5) (dashed line). The fit is of a reasonable quality, with *R*^2^ value of 0.87. The Poissonian fit in Figure 6 is distinctly different from the energy transfer efficiency predicted by the Förster theory (Equation (3)) using the *R*_0_ and *R*_DA_ values of 7.13 and 6.7 nm, respectively (solid line). 

**Figure 7:**
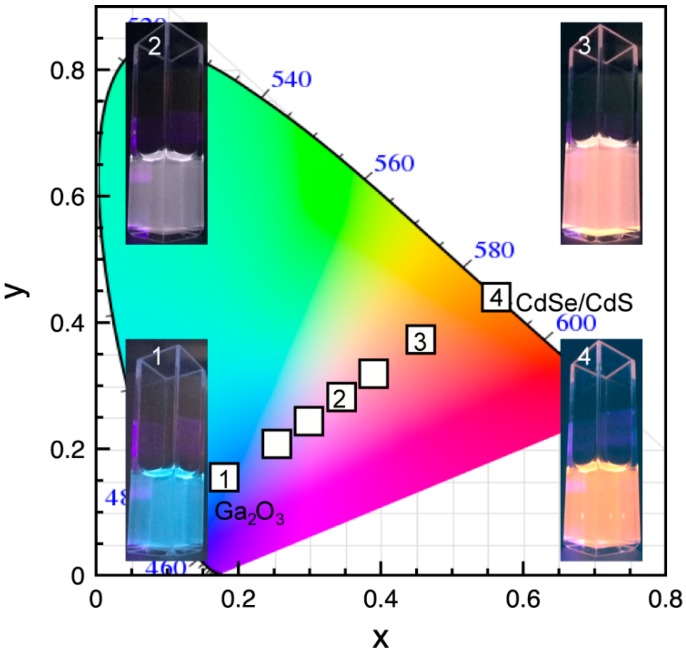
International Commission on Illumination 1931 (CIE 1931) color coordinate diagram for Ga_2_O_3_-CdSe/CdS NC conjugate having different acceptor to donor ratio. The photographs of the colloidal nanoconjugates corresponding to data points labeled in the graph are shown as insets.

The ability to control FRET efficiency in Ga_2_O_3_-CdSe/CdS NC conjugates by adjusting the acceptor to donor ratio can be used to control the overall chromaticity of the emitted light. Figure 7 shows an International Commission on Illumination 1931 (CIE 1931) color space diagram of the NC conjugates consisting of different average number of acceptors per donor. The photographs of the colloidal suspensions of the conjugates corresponding to the symbols labeled in the graph are shown as insets. The non-conjugated Ga_2_O_3_ NCs emit blue light (inset 1). The addition of CdSe/CdS NCs increases the orange-red contribution to the spectral power distribution, owing to both energy transfer and direct excitation. For an optimal ratio of CdSe/CdS to Ga_2_O_3_ NCs the colloidal NC conjugate suspension has color coordinates in the white light emitting region (0.345, 0.282) (inset 2). The orange-red contribution becomes dominant for high concentration of the CdSe/CdS NCs (inset 3), reaching bright orange color for pure CdSe/CdS NC suspension (inset 4). The CIE 1931 diagram for the mixtures of Ga_2_O_3_ and CdSe/CdS NCs is shown in Figure S4 (Supplementary Materials) for comparison. These results demonstrate the promise of using inorganic building blocks to generate tunable white light with characteristic spectral and color properties.

## 3. Materials and Methods

### 3.1. Materials

All materials are commercially available, and were used as received. Gallium acetylacetonate (Ga(acac)_3_) was purchased from Strem Chemicals (Newburyport, MA, USA). Cadmium oxide, selenium, 1-octadecene, trioctylphosphine (TOP), oleylamine, oleic acid, thioglycolic acid (TGA), trioctylphospine oxide (TOPO), tetradecylphosphonic acid, and tetrahydrofuran (THF) were all purchased from Sigma Aldrich (St. Louis, MO, USA).

### 3.2. Synthesis of Ga_2_O_3_ NCs

The synthesis of Ga_2_O_3_ NCs was carried out according to the previously reported method [16]. Briefly, 0.5 g of Ga(acac)_3_ and 14 g of oleylamine were loaded into a 100 mL three-neck round bottom flask. The reaction flask was filled with argon and heated to 300 °C over the course of an hour. The reaction mixture was held at this temperature for an hour, and then cooled naturally to room temperature. The obtained product was washed with ethanol and centrifuged at 3000 rpm. The washing procedure was repeated three more times. The NCs were finally capped with TOPO, as previously described [16]. The capped NCs were dispersed in THF.

### 3.3. Synthesis of CdSe NCs

CdO (60 mg), tetradecylphosphonic acid (210 mg), and TOPO (3 g) were combined in a 100 mL three-neck round bottom flask. The flask was degassed at 150 °C for 1 h, then back filled with argon. The reaction mixture was brought to 320 °C and 1 mL of TOP-Se, prepared separately by dissolving 60 mg Se in 1 mL TOP, was injected rapidly. The reaction was allowed to proceed until the desired NC size is achieved, then cooled using compressed air. When the temperature reached 80 °C methanol was injected, and the precipitated NCs were isolated by centrifugation at 3000 rpm. The NCs were then dispersed in hexane, and their concentration was determined from the absorption spectra [21].

### 3.4. CdS Shell Growth

The growth of CdS shell was performed by a previously reported method [22]. In summary, as-synthesized suspension of CdSe NC cores (*ca*. 100 nmol) was placed in a three-neck round bottom flask containing 3 mL oleylamine and 3 mL 1-octadecene. The reaction flask was held under vacuum for 1 h and subsequently purged with nitrogen. The shell was grown from Cd(OA)_2_ and dodecanethiol as precursors. Cd(OA)_2_ was prepared by dissolving 1 part CdO in 4 parts oleic acid at 230 °C, while dodecanethiol was dissolved in 1-octadecene. The temperature of the flask containing NC cores was increased to 320 °C, and the dropwise injection of 6 mL Cd(OA)_2_ and dodecanethiol solutions started at 240 °C. Injection was carried out for 3 h, after which 3 mL of oleic acid was injected and the colloidal mixture was further heated for 1 h. The reaction mixture was cooled and washed with acetone, and dissolved in THF.

### 3.5. Functionalization of CdSe/CdS NCs with TGA

The capping of CdSe/CdS NCs with TGA was carried out by combining 1 mL of 5 μM CdSe/CdS NCs in hexanes, 1 mL acetone, and 0.2 mL TGA in a 25 mL scintillator vial under a nitrogen atmosphere, and stirring for 30 min. 0.5 mL ethanol was added to the vial and the mixture was stirred for another 15 min. The product was isolated by centrifuging at 3000 rpm. The precipitate was washed again in ethanol, and the supernatant liquid was collected. This process was repeated once more, and the resulting TGA-capped NCs were dispersed in THF. The amount of unbound TGA linker in the supernatant was determined by absorption spectroscopy, using a calibration curve established by measuring the absorbance of TGA solutions of known concentrations.

### 3.6. Preparation of the Nanoconjugate

One milliliter of stock suspension of Ga_2_O_3_ NCs in THF was placed in a 25 mL vial. Varying amounts of TGA-bound CdSe/CdS NCs (0–1 mL stock suspension) were added to these vials, and the mixture was diluted with additional THF to the final volume of 2 mL. These suspensions were left for 4 h and then characterized spectroscopically.

### 3.7. Spectroscopic Measurements

Absorption spectroscopy measurements were performed with a Varian Cary 5000 UV-vis-NIR spectrophotometer (Agilent Technologies, Santa Clara, CA, USA) using standard 1 cm path length quartz cuvettes. Photoluminescence spectra were collected with a Varian Cary Eclipse fluorescence spectrometer. Time-resolved PL measurements were performed with a Horiba Jobin Yvon iHR320 time-correlated single photon counting spectrometer (Edison, NJ, USA) using a 249 nm NanoLED and a Horiba Jobin Yvon TBX picosecond detector.

## 4. Conclusions

In summary, we demonstrated a new white light-emitting colloidal NC conjugate consisting of CdSe/CdS core/shell NCs connected to γ-Ga_2_O_3_ NCs via TGA. Owing to the short chain of TGA molecule, the NC conjugate is a donor-acceptor system undergoing FRET, where Ga_2_O_3_ NCs act as energy donors and CdSe/CdS NCs as energy acceptors. In spite of the direct excitation of the acceptor NCs, the FRET mechanism is evident through quenching of the donor PL, shortening of the donor emission lifetime, and an increase in the acceptor emission. The control of the CdSe/CdS NC excitation by simultaneous light absorption and energy transfer from Ga_2_O_3_ NCs allows for the control of the overall emission chromaticity of the NC conjugate, including the generation of the white light. The results of this work represent a step toward the design of all-inorganic white light emitting phosphors for various lighting applications.

## Supplementary Materials

The following are available online at http://www.mdpi.com/2079-4991/6/2/32/s1. Figure S1: deconvoluted PL spectra of CdSe/CdS NCs, Figure S2: absorption spectrum of TGA, Figure S3: excitation spectra of CdSe/CdS NCs in the NC conjugate, Figure S4: CIE 1931 color space diagram for mixtures of non-linked CdSe/CdS and Ga_2_O_3_ NCs.
