# Supplementary material for: Energy Transfer between Conjugated Colloidal Ga2O3 and CdSe/CdS Core/Shell Nanocrystals for White Light Emitting Applications
Source: Nanomaterials (Basel). 2016 Feb 15;6(2):32. doi: 10.3390/nano6020032 (PMC5302483; doi:10.3390/nano6020032)
Supplement: Supplementary file 1 [file nanomaterials-06-00032-s001.pdf]

Supplementary Materials: Energy Transfer between Conjugated Colloidal Ga_2O_3 and CdSe/CdS Core/Shell Nanocrystals for White Light Emitting Applications

Paul C. Stanish and Pavle V. Radovanovic

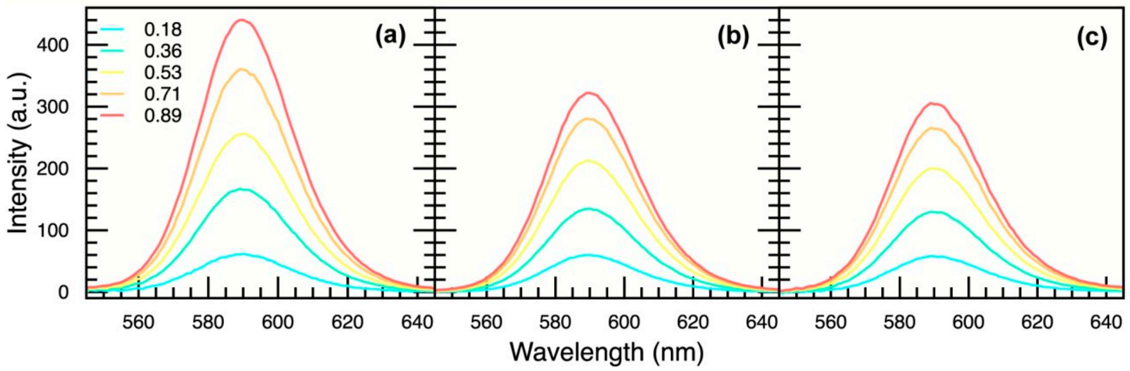

Figure S1. Photoluminescence spectra of CdSe/CdS nanocrystals (NCs) in (**a**) nanocrystal conjugate, linked with thioglycolic acid (TGA), upon correction for the contribution from Ga_2O_3 NC emission, (**b**) pure CdSe/CdS nanocrystal suspension, and (**c**) mixture of Ga_2O_3 and CdSe/CdS NCs without the TGA linker. The concentrations of CdSe/CdS NCs are identical in all panels, and are expressed in (**a**) as a [CdSe/CdS]/[Ga_2O_3] ratio. The excitation wavelength is 250 nm for all spectra.

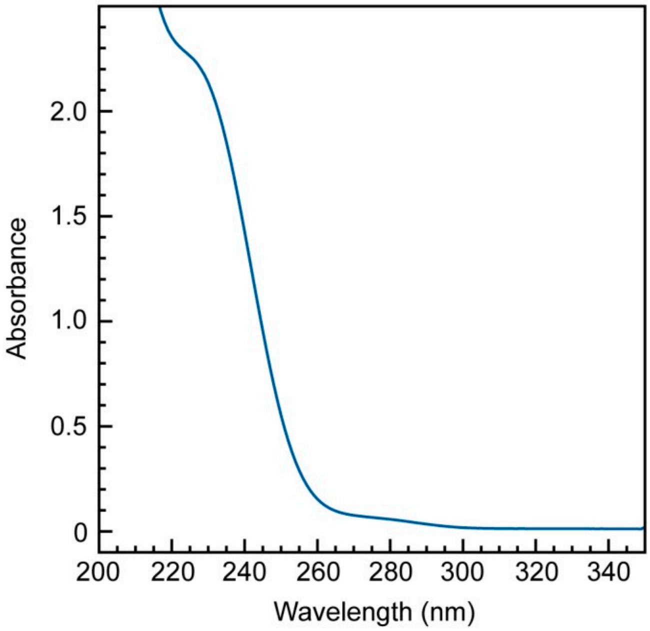

Figure S2. Absorption spectrum of TGA molecules in ethanol demonstrating competitive absorption with Ga_2O_3 NCs at 250 nm.

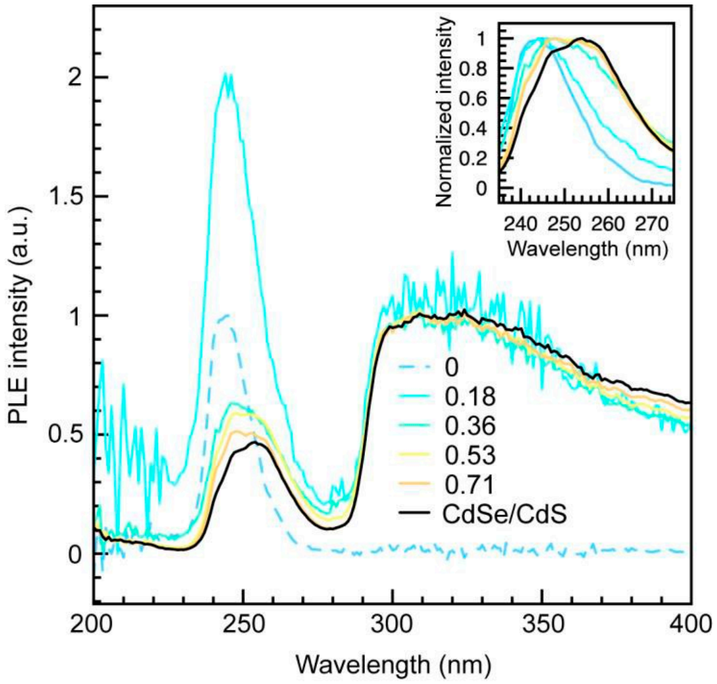

Figure S3. Photoluminescence excitation (PLE) spectra of CdSe/CdS-Ga_2O_3 NC conjugates having different acceptor to donor ratio, as indicated in the graph (λ_{em} = 590 nm). PLE spectra of Ga_2O_3 and CdSe/CdS NCs are shown with dashed blue and solid black lines, respectively. Inset: Normalized PLE spectra in the region corresponding to Ga_2O_3 NC excitation. The dip in the excitation spectra at *ca*. 280 nm is due to absorption of tetrahydrofuran (THF) in that region.

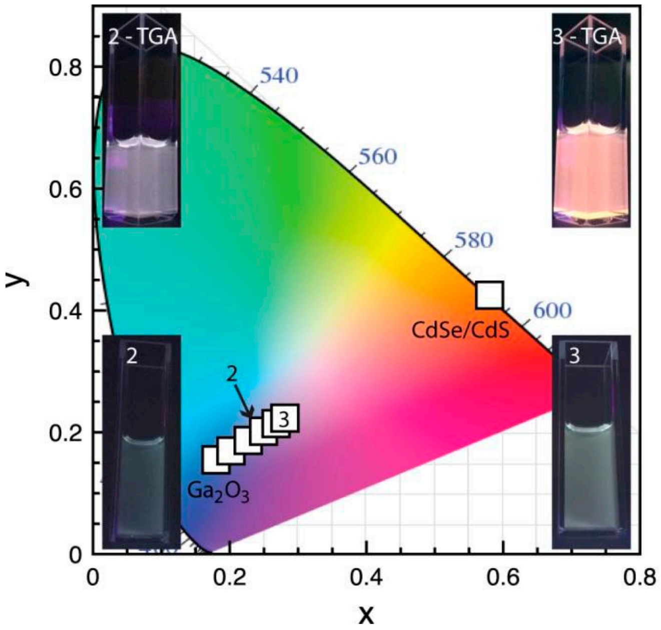

Figure S4. International Commission on Illumination 1931 (CIE 1931) color space diagram for suspensions containing a mixture of CdSe/CdS and Ga_2O_3 NCs. The mixtures are prepared in an identical way to the samples shown in Figure 7 in the article, but without the TGA linker. Bottom insets show photographs corresponding to points 2 and 3 in the graph. The photographs of analogous samples for the NC conjugates are shown as top insets for comparison.

© 2016 by the authors; licensee MDPI, Basel, Switzerland. This article is an open access article distributed under the terms and conditions of the Creative Commons by Attribution (CC-BY) license (http://creativecommons.org/licenses/by/4.0/).
